# A Comparison of Interpolyelectrolyte Complexes (IPECs) Made from Anionic Block Copolymer Micelles and PDADMAC or q-Chitosan as Polycation

**DOI:** 10.3390/polym15092204

**Published:** 2023-05-06

**Authors:** Özge Azeri, Dennis Schönfeld, Bin Dai, Uwe Keiderling, Laurence Noirez, Michael Gradzielski

**Affiliations:** 1 Stranski-Laboratorium für Physikalische und Theoretische Chemie, Institut für Chemie, Technische Universität Berlin, D-10623 Berlin, Germany; 2 Helmholtz-Zentrum Berlin (HZB) für Materialien und Energie GmbH, 14109 Berlin, Germany; 3 Laboratoire León Brillouin CEA-CNRS, Université Paris-Saclay, F-91191 Gif-sur-Yvette, France

**Keywords:** interpolyelectrolyte complexes (IPECs), amphiphilic copolymers, atom transfer radical polymerization (ATRP), chitosan, ionic assembly, small angle neutron scattering (SANS)

## Abstract

Block copolymers synthesized via Atom Transfer Radical Polymerization from alkyl acrylate and t-butyl acrylate and the subsequent hydrolysis of the t-butyl acrylate to acrylic acid were systematically varied with respect to their hydrophobic part by the variation in the alkyl chain length and the degree of polymerisation in this block. Depending on the architecture of the hydrophobic part, they had a more or less pronounced tendency to form copolymer micelles in an aqueous solution. They were employed for the preparation of IPECs by mixing the copolymer aggregates with the polycations polydiallyldimethylammonium chloride (PDADMAC) or q-chit. The IPEC structure as a function of the composition was investigated by Static Light and Small Angle Neutron Scattering. For weakly-associated block copolymers (short alkyl chain), complexation with polycation led to the formation of globular complexes, while already existing micelles (long alkyl chain) grew further in mass. In general, aggregates became larger upon the addition of further polycation, but this growth was much more pronounced for PDADMAC compared to q-chit, thereby leading to the formation of clusters of aggregates. Accordingly, the structure of such IPECs with a hydrophobic block depended largely on the type of complexing polyelectrolyte, which allowed for controlling the structural organisation via the molecular architecture of the two oppositely charged polyelectrolytes.

## 1. Introduction

Mixtures of oppositely charged polyelectrolytes in an aqueous solution typically lead to the formation of interpolyelectrolyte complexes (IPECs) [1]: a process that is driven by the release of counterions [2]. IPECs are promising for a number of applications, such as in medicine and agricultural biotechnology [3], because of their rather variable options for solubilisation and binding. They have been studied for a long time, initially mostly for oppositely charged homopolyelectrolytes, which tended to form insoluble complexes around a charged equilibrium and soluble complexes for a significant excess of polycation or polyanion [4]. However, one may also form more complex built IPECs by combining ionic block copolymers possessing a second hydrophobic block and oppositely charged polyelectrolytes. Such copolymer micelles are more complex colloidal aggregates that may contain a purely hydrophobic core, which is then surrounded by an IPEC shell, and, still further outside, one has a corona of the excess polyelectrolyte that stabilizes such polymer colloids in an aqueous solution. They are interesting as their hydrophobic core may be able to solubilize a hydrophobic cargo, and the IPEC shell then is a way to separate them from the aqueous surrounding and/or to allow for their controlled release from the core. In addition, the IPEC shell may itself be the location of the selective solubilization of more polar compounds. Accordingly, copolymer micelle IPECs are interesting systems, and due to the large variety of copolymers and polyelectrolytes available, as well as the possibility of varying their Molecular Weight (Mw) over a large range, there are almost endless options available to form them by an appropriate combination of components. In this way, one is also able to tune structures and properties over a very wide range and to produce colloids of tailor-made properties.

Copolymer micelle IPECs have already been reviewed some a while ago [1] and frequently show properties that strongly depend on the stoichiometry and sequence of mixing, as well as a marked asymmetry in solubility as a function of the mixing stoichiometry [5]. An important aspect is also the thermodynamic state of the hydrophobic core, as its hydrophobic part may be above the glass transition temperature and, therefore, in a fluid and rather liquid state that equilibrates quickly when external conditions, such as pH or ionic strength, are changed [6] or are below the glass transition temperature, for which these hydrophobic cores are in a glassy state and the formed micelles can be considered frozen [7]. For this situation, an equilibration may effectively never be achieved, but it has also been observed that equilibration may be very slow and proceed via the intermediate formation of clusters of such frozen micelles bridged together via the oppositely charged polyelectrolyte [8]. Simpler is the case with fluid hydrophobic cores, as it has, for instance, been realised for the case of polyisobutylene/poly (methacrylic acid) block copolymers. They form anionic micelles, which can become complexed by the strong polycation poly(N-ethyl-4-vinylpyridinium bromide) (PVP EtBr) to yield stable IPEC micelles up to a charge ratio z = [+]/[−] of 0.4, while at a higher concentration of polycation, macroscopic precipitation can be observed [9]. In general, the dynamic response of such complexes depends on the steric and electrostatic interactions between the chains and, correspondingly, can vary largely for such different systems [10].

One of the most studied polyelectrolytes is PDADMAC which has a positive charge in aqueous solutions stemming from quaternary ammonium groups, which can be used in paper manufacturing [11], the mining industry [12], and water treatment [13,14]. Queirós and Loh [15] worked on the phase behaviour of the interpolyelectrolyte complex (IPEC) coacervates of poly(acrylate) (PA) and PDADMAC in the presence of inorganic salts. Titrations of polyelectrolytes in their acidic and alkaline forms were performed to obtain coacervates in the absence of their small counterions. This study was performed with respect to two different parameters. One parameter was the molar mass of the PA, ranging between 2 and 100 kDa, and the second parameter was the type of added salt as NaCl and Na_2_SO_4_. It was observed that the IPEC with the larger molar mass PA_100kDa_ was more stable than the one with a smaller molar mass PA_2kDa_. Moreover, the authors stated that the complex of PDADMAC and PA_2kDa_ was more stable toward the addition of NaCl than Na_2_SO_4_. This could be interpreted in terms of charge screening because of the presence of electrolytes, which are expected to become stronger as the ion valency increases [16].

Jemili et al. [17] investigated the complexation between hydrophilic PDADMAC, hydrophobic polyanion, and polystyrene-co-sodium styrene sulfonate with varying degrees of sulfonation (P(ST-co-SSNa)) using techniques such as TEM, AFM, ITC, DLS and spectrophotometric titration. They found that stable IPECs were formed on both sides of the charge neutrality, while rather unstable IPECs with low zeta potential were formed close to the charge neutrality. Moreover, the results showed the existence of a two-step complexation mechanism. The first step was the generation of ion pairs between oppositely charged polyelectrolytes (PE) with sizes below 50 nm due to the attractive electrostatic interactions. The aggregation of these primary PECs into bigger clusters is the second step. Additionally, the size of the IPECs ranges from 100 to 300 nm as determined by the dynamic light scattering experiments (DLS), which do not depend on the sulfonation rate of P(St-co-SSNa).

Lately, in our group, Kuzminskaya et al. [18] reported a study about interpolyelectrolyte complexes of PDADMAC and PA, which mainly focused on the effect of the hydrophobic modification of PA on the structure and phase behaviour of complexes formed with PDADMAC, covering a wide range of molecular weight. The hydrophobic modification of PA was obtained by having 10 mol% monomeric units substituted by ones from a dodecyl alkyl acrylate chain. The complexes were characterized via DLS, SLS and SANS to obtain detailed information on the structure of the soluble complexes. It was found that the hydrophobic modification led to phase separation for lower amounts of added polyanion, and the biphasic region was larger than the complexes with unmodified PA. Moreover, light scattering experiments proved that their complexes became larger upon approaching a charged equilibrium. SANS data of the complexes showed that these complexes tended to form clusters of aggregates with sizes of around 35 nm with smaller subunits of 2.5–5.0 nm in size.

Another commonly used polymer was Chitosan which is a cationic polysaccharide [19] and has received increasing attention in recent decades due to its unique biocompatibility, biodegradability, non-toxicity [20] and medical properties [21]. Accordingly, chitosan has also been widely applied in the field of polyelectrolyte complex formation, as reviewed some while ago [22]. Chen et al. [23] studied the structure of the IPECs composed of chitosan and poly(methacrylic acid) (PMAA) in aqueous solutions by means of UV-vis spectroscopy, a fluorescence probe technique, and transmission electron microscopy (TEM). The results showed that for pH 4.0 and a molar ratio of Chitosan to the PMAA of 1:4, well-defined complexes of Chitosan and PMAA were formed. For pH > 4.0, the degree of ionization of PMAA increased, but that of Chitosan decreased. Moreover, TEM results showed that the complex particles exhibited a very compact spherical structure. The size of the particles at pH = 3.0 was between 10 and 17.5 nm; at pH = 4.0, it was between 22.5 and 40.0 nm and then increased to about 75 nm for pH = 4.5.

Carvalho et al. [24] investigated the influence of pH, molecular weight, and polymeric proportion on the formation of IPECs based on chitosan: dextran sulfate. It was stated that nanoparticles in the polycation-rich regime formed aggregates, while an excess of dextran sulfate reduced the size of the particles. Sæther et al. [25] studied IPECs of alginate and chitosan using a one-stage process under high shearing conditions. They mainly focused on how the IPEC particle sizes and surface charge could be controlled by varying preparation procedures and polymer characteristics. These complexes were prepared with varying rates and diameters of the dispersing elements of the homogenizers to examine the effect upon the IPECs formed. It was found that the size of the complexes decreased with the increasing rate of homogenization. Additionally, the results showed that polyelectrolyte complexes made from chitosans and alginates with low molecular weights formed smaller complexes in comparison to that with high molecular weights.

Lima et al. [26] published a study about the formation and structure of IPECs composed of chitosan and poly(sodium methacrylate), which was produced by mixing solutions at different carboxyl-to-amine molar ratios, r*_CA_*. The small angle X-ray scattering (SAXS) experiments proved that at r*_CA_* = 0.15, the structure of formed aggregates was nearly spherical. When r*_CA_* was raised up to 0.75, the Kratky plot indicated the presence of elongated structures. For r*_CA_* > 0.75, these structures had a tendency to collapse back to nearly spherical ones. Despite particle structures becoming more elongated at r*_CA_*= 0.75, the radius of gyration markedly dropped to 6 nm, exhibiting the occurrence of collapse at this ratio.

Accordingly, complexes of chitosan or PDADMAC and polyacrylate have been studied to quite some extent. However, so far, only a few studies have been conducted using quaternized chitosan, which showed the slight pH dependence of its complexation properties. Similarly, the complexation of the diblock copolymer of polyacrylate with an additional hydrophobic block has been studied very little. Closing this gap was the centre of our work, in which we focused on amphiphilic block copolymers of the alkyl acrylate-sodium acrylate (AlkA-NaPA) type. Such systems have been described before and showed the formation of well-defined micelles [27]. More recently, we studied the aggregation behaviour of AlkA-NaPA copolymers, where we varied the alkyl rest from butyl to dodecyl, i.e., the extent of hydrophobicity of the micellar core. This study by critical micelle concentration (cmc) determination and light and neutron scattering showed that the tendency for micellization and the size of the formed micelles depended in a systematic way on the length of the alkyl chain of the acrylate and on the total length of the hydrophobic block [28]. This type of copolymeraggregate, where the internal polarity is controlled by the type of alkylacrylate and the size of the hydrophobic domains are determined by the length of their polymeric unit, was then employed to form IPECs with quaternized chitosan (q-chit) and for comparison also with the well-studied synthetic polycation PDADMAC [29]. This is interesting as, in that way, one can create highly versatile polymeric colloids with a hydrophobic core of variable polarity and size, surrounded by an IPEC shell, which is highly polar, though water-insoluble, and has an affinity for solubilisation that depends on the precise composition of the IPEC. By employing polycations with a different type of chain backbone for complexation, there was an option for varying the IPEC structure, and by using q-chitosan, one had, in addition, a biopolymer that could lead to an overall better biocompatibility of the formed aggregates. It might be noted that we chose the Mw of the polycations, such that their stretched length was rather equal and in the range of ~450 nm (see Supplementary Materials Section S5).

In our investigation, we worked at a constant concentration of copolymer micelles and increased the content of added polycation. For the copolymer, we employed hydrophobic blocks of butyl acrylate, hexyl acrylate, and dodecyl acrylate at different lengths (40–70 units) and as hydrophilic block sodium polyacrylate (with 70–170 units). These copolymers had been shown before to vary largely with respect to their tendency for forming micelles, which is very pronounced for dodecyl and rather weak for butyl chains [28]. In the experiments described here, we studied the phase behaviour and, in particular, the structure of the aggregates formed upon complexation with oppositely charged polycations, which were expected to bind to the surface of the polymeric micellar aggregates. These structural studies were conducted by means of light and neutron scattering.

## 2. Materials and Methods

### 2.1. Materials

The anionic amphiphilic alkyl acrylate-sodium acrylate (AlkA-NaPA) block copolymers were synthesized as described before, and their molecular characterization was also given in a previous publication [28]. Poly(diallyldimethylammonium chloride) PDADMAC (M_w_: ~150 kDa) was purchased from Sigma Aldrich and used after drying in a freeze dryer without any further purification. The second polycation was quaternised chitosan (q-chit; M_w_: ~180 kDa) synthesized in our group by a modified version of a synthesis previously described [30,31]. This product had a degree of quaternisation of 0.785, a degree of acetylation of 0.185, and a degree of O-methylation of 0.17. The characteristic parameters for all polyanions employed are summarized in Table 1, where they have abbreviated afterward according to the type and length of the hydrophobic block and for the dodecyl copolymer in addition to “s” for the short and “l” for the long poly(acrylic acid) (PAA) block, respectively.

Milli-Q water was produced by a Millipore filtering system. D_2_O was obtained from Eurisotop (99.5% isotopic purity, Gif-sur-Yvette, France). Sodium hydroxide (99%) and sodium chloride (>99%) were obtained from Sigma-Aldrich (Taufkirchen, Germany). Toluene (>99.5%) from Fluka, methyl-2-bromopropionate (2-MBP, 98%) and hexane from Aldrich (Steinheim, Germany), N,N,N′,N″,N″-pentamethyldiethylenetriamine (PMDETA, 99%), hexyl acrylate (98%), dodecyl acrylate (technical grade 90%) from Sigma-Aldrich and diethylether (>99.5%) from Carl-Roth GmbH (Karlsruhe, Germany) were used as supplied. *tert*-butylacrylate, *n-*butylacrylate and dichloromethane were gifts from BASF (Ludwigshafen, Germany) and were used as supplied.

### 2.2. Preparation of Complexes

All samples were prepared in H_2_O or D_2_O. Before the preparation of the samples, D_2_O was filtered by passing through a PTFE filter with a 0.45 µm pore size in order to obtain dust-free samples. The degree of deprotonation for the stock solution of polyanions was adjusted to α = 1.0 (formally 1.2, as a slight excess of 20% of NaOH was added to ensure complete deprotonation). The stock solutions were prepared with a concentration of 10 g/L for polyanions and 15 g/L for polycations. The charge ratio, z, was defined as [+]/[−] and was changed from 0 to 0.4. The concentration of polyanion in the complexes was kept constant at 5 g/L for all the samples. First, the required amount of polyanion was added to a glass vial. Then, the solvents, H_2_O or D_2_O, were added into the vial to obtain the appropriately diluted solution before adding the polycation solution. As a last step, the stock solution of polycation was added dropwise under strong stirring in order to avoid any precipitation due to the local neutralization of the sample.

### 2.3. Methods

Zeta-potential measurements were performed on a Litesizer 500 by Anton Paar GmbH (Graz, Austria) at 25 °C. Zeta-potential ζ was calculated from the measured electrophoretic mobility μE as:

(1) $$\zeta =\frac{3\eta \mu_{E}}{2\varepsilon_{0\;}\varepsilon_{r\;}},\;$$

where *η* is the viscosity. $\varepsilon_{0\;}$ is the permittivity of the vacuum and $\varepsilon_{r\;}$ is the relative permittivity of the medium.

The kinematic (*ν*) viscosities were measured with the automated viscosimeter iVisc, and Lauda Scientific GmbH (Lauda-Königshofen, Germany) using calibrated Micro-Ostwald capillary viscometer (Typ Ic-II) at a constant temperature of (25.0 ± 0.1) °C. One pre-measurement and five main measurements were performed. The dynamic (*η*) viscosities were calculated after density measurements at (25.0 ± 0.1) °C (DMA4500, Anton Paar GmbH). The samples were prepared in heavy water, *D*_2_*O.*

According to Beer–Lambert Law, the transmission, T, can be related to the attenuation coefficient (or turbidity), τ, [32]

(2) $$T=\frac{I_{L}}{I}=\exp\left(-\tau d\right),\;$$

where $I_{L}$ is the intensity of the beam traversing the sample of thickness *d* and *I* is the incident intensity.

The transmission of the samples was measured with a Cary 50 spectrometer by Varian in a UV-Vis cuvette of thickness *d* = 10 mm or Quartz cuvettes with *d* = 2 or 1 mm.

The apparent molecular weight ($M_{w,\lambda }^{app})$ of the complexes was calculated from transmittance measurements at 632.8 nm according to:

(3) $$M_{w,\lambda }^{app}=\frac{\tau_{\lambda }}{H_{0,\lambda }\cdot c}=\frac{\;3N_{Av}\cdot \lambda^{4}}{32\pi^{3}\cdot n_{0}^{2}\cdot {\left(\frac{dn}{dc}\right)}_{\lambda }^{2}\cdot c_{g}}\cdot \tau$$

The optical constant was given by:

(4) $$H_{0,\lambda }=\frac{32\pi^{3}\cdot n_{0}^{2}\cdot {\left(\frac{dn}{dc}\right)}_{\lambda }^{2}}{3N_{Av}\cdot \lambda^{4}}$$

where *N_Av_* is the Avogadro constant, *n*_0_ is the refractive index, *dn*/*dc* is the refractive index increment; *c* is the mass/volume concentration, and *λ* is the wavelength of light.

Static light scattering (SLS) experiments were carried out with a CGS-3 (compact goniometer system) and a HeNe-Laser at 632.8 nm wavelength from ALV GmbH (Langen, Germany). Two avalanche photodiodes (APD) were used to detect the scattered light in an angular range of 40–140°.

For the static light scattering experiments, the measured intensity had to be corrected and normalized for each angle as given by Equation (5).

(5) $$I\left(q\right)=\frac{{\left(\frac{CR1}{I_{mon}}\right)}_{sample}-{\left(\frac{CR1}{I_{mon}}\right)}_{solvent}}{{\left(\frac{CR1}{I_{mon}}\right)}_{toluene}}*R_{\theta ,toluene}$$

*CR*1 is the mean scattered intensity of the samples, normalized by the initial laser intensity $I_{mon}$. The background scattering of the solvent and cuvette was subtracted, and a calibration with toluene was applied, where $R_{\theta ,toluene}$ is the Rayleigh ratio of toluene [33].

The scattered intensity *I*(*q*) for particles should exhibit an angular dependence according to Guinier’s law:

(6) $$I\left(q\right)=I\left(0\right)\;•\;exp\;\left(-\frac{q^{2}R_{g}^{2}}{3}\right)\;$$

(7) $$q=\frac{4\pi n_{0}\sin\theta /2}{\lambda }$$

where *Rg* is the radius of gyration, *q* is the modulus of the scattering vector, *θ* is the scattering angle, and *λ* is the wavelength of the light. From the intensity at a zero angle, *I*(0), one can, via the optical constant K:

(8) $$K=4\frac{\pi^{2}*n_{0}^{2}*{\left(\frac{dn}{dc}\right)}^{2}}{N_{Av}*\lambda^{4}}$$

directly calculate the apparent molecular weight of the scattering objects, $M_{w}^{app}$

(9) $$M_{w}^{app}=\frac{I\left(0\right)}{c\;*\;K}$$

where *dn*/*dc* is the refractive index increment. The refractive index increment (*dn*/*dc*) of the synthesized polymers was measured with the instrument Orange 19″ DN/DC (see Supplementary Materials Section S4).

SANS measurements were performed at a PA20 spectrometer of the Laboratoire Léon Brillouin (LLB, Saclay, France). Three configurations were used with 1.9, 8.3, and 18.8 m sample-to-detector distances (SD) and a wavelength of 6 Å. To reach a higher q, we used an off-centred detector position at the shortest detector distance, 1.9 m. In the experiments, a q-range of 2.5 × 10^−2^ to 3.2 nm^−1^ was covered. Some additional SANS measurements were conducted on the V4 instrument at Helmholtz Zentrum Berlin (HZB, Berlin, Germany). Three configurations were used: with an SD and collimation (C) of: 1.35 m (SD) and C = 8 m, 8 m (SD), C = 8 m, 15.60 m (SD) and C = 16 m. Two wavelengths of 4.5 and 12 Å (for SD = 15.60 m) were employed. In these experiments, a q-range of 2.3 × 10^−2^ to 6.4 nm^−1^ was covered.

The coherent scattering intensity was obtained after normalization of the detector cell efficiency using an incoherent scatterer (H_2_O), the subtraction of empty cell scattering and electronic noise (Cd). The scattering curve was obtained by isotropic re-groupment with respect to the scattering centre, and, taking into account the transmissions, the differential cross-sections were calculated [34]. All data evaluation was conducted using the BerSANS (Aug 2014) software [35]. Subsequently, the data sets obtained for the three different configurations were merged. Data analysis was performed with SasView (5.0.5) an open-source scattering analysis software [36].

SLD of the complexes ($SLD_{comp}$) was calculated from the sum of the volume fractions Φi of the polyanion or the polycation multiplied with the SLD of the corresponding part (i) (see Supplementary Materials Section S6.1).

## 3. Results

### 3.1. Phase Behaviour

**Figure 1:**
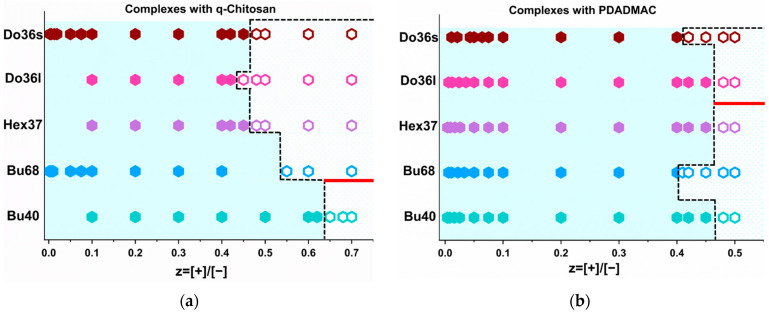
Phase behaviour of the complexes with (**a**) q-chit and (**b**) PDADMAC at a fixed concentration of polyanion of 5 g/L and at T = 25 °C. The light blue area points out the 1-phase region which is separated from 2-phase region with a dashed line. For open symbols for 2-phase region, the formation of precipitates in the biphasic region is indicated under the red line and that of coacervates above the red line.

As IPECs are known to form precipitates or coacervates around equimolar charge conditions, we first studied the phase behaviour of the different block copolymer micelles at a constant concentration of 5 g/L upon the addition of increasing amounts of polycation. The result is shown in Figure 1, and for PDADMAC, the phase boundary was always in the range of z ~ 0.45. For q-chit, it this similar for the more hydrophobic copolymers with dodecyl and hexyl acrylate, but for the butyl acrylate, the phase boundary was shifted to a larger z and increasingly so for a lower degree of polymerisation of the butyl acrylate. Interestingly, phase behaviour was more sensitive for q-chit as a complexing polycation, and this followed the expected trend of higher solubility with the decreasing hydrophobicity of the copolymer.

Another interesting point is that complexes with PDADMAC at z ~ 0.45 precipitated for a relatively shorter alkyl chain-containing polyanions, i.e., Bu40, Bu68 and Hex37, while the complexes with dodecyl-containing polyanions formed coacervates.

Apparently, for the latter, the formation of hydrophobic domains did not allow the formation of a compacted structural arrangement, and instead, a marked swelling of the systems took place. For the complexes with q-chit at z = 0.65, precipitates were only formed with the least hydrophobic polyanion, Bu40, while coacervation took place at z = 0.55 for Bu68 and z = ~0.45 for the complexes with Hex37, Do36l and Do36s was observed after preparation. This indicated that q-chit enhanced the tendency for swelling with water, which may have been due to the OH-groups it carried along its backbone.

The zeta potential of IPECs with the most and the least hydrophobic polyanions was measured and showed a similar behaviour upon complexation with PDADMAC or q-chit. However, the addition of q-chit led to a faster increase in zeta-potential (Table S1) which at first glance was surprising as the q-chit formed more stable complexes, i.e., the ones where a higher z still colloidally stable solutions were obtained (Figure 1). However, it also indicated that q-chit more effectively neutralised and bonded to the anionic block copolymer aggregates, which is in good agreement with its tendency not to form clusters of these aggregates, as discussed later.

### 3.2. Turbidity Measurements

**Figure 2:**
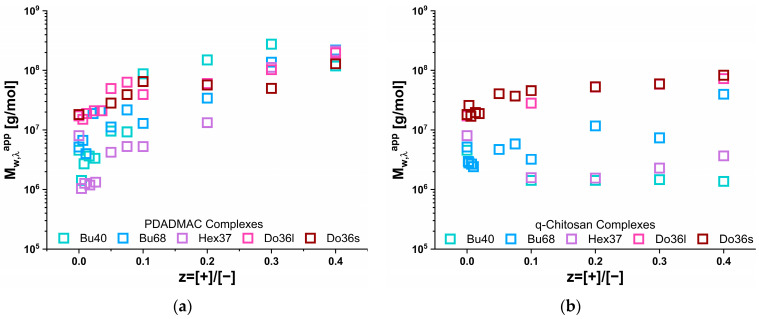
Apparent molecular weight ($M_{w,\lambda }^{app}$) of the IPECs obtained by complexing solutions of 5 g/L AlkA-b-NaPa with different amounts of polycation (**a**) PDADMAC or (**b**) q-chit from turbidity measurements (at 632.8 nm).

The apparent molecular weight ($M_{w,\lambda }^{app})$ of the complexes was calculated from the turbidity (*τ*) of the samples. In doing so, quite different behaviour for the complexation with PDADMAC and q-chit was found, as shown in Figure 2. For q-chit $M_{w,\lambda }^{app}$ changed only a little, mostly increasing somewhat with increasing z, but for the case of Hex37 and Bu40, even decreasing slightly compared to the pure copolymer micelles always formed rather small aggregates. This meant that the micelles became somewhat complexed by the q-chit and also partly even rearranged in this neutralisation process, thereby explaining the rather constant, partly even reducing $M_{w,\lambda }^{app}$. Clearly, the largest complexes were formed with the dodecyl acrylate, which, being the most hydrophobic of the alkyl acrylates employed, also formed the most well-defined copolymer micelles. Bu68 showed a smaller but consistent increase in $M_{w,\lambda }^{app}$.

This situation was quite different for complexation with PDADMAC, where already low z values up to 0.05 and a substantial increase in $M_{w,\lambda }^{app}$ by a factor of 10–100 took place. For a still higher z value, the increase continued but at a smaller rate. The correlation between the type of hydrophobically modified polyanion and the $M_{w,\lambda }^{app}$ hardly existed. Only at the lowest z were the highest values for Do36s and Do36l found, but with increasing z, the differences became smaller, and all complexes at z = 0.4 exhibited values of 1–2⸱10^8^ g/mol.

**Figure 3:**
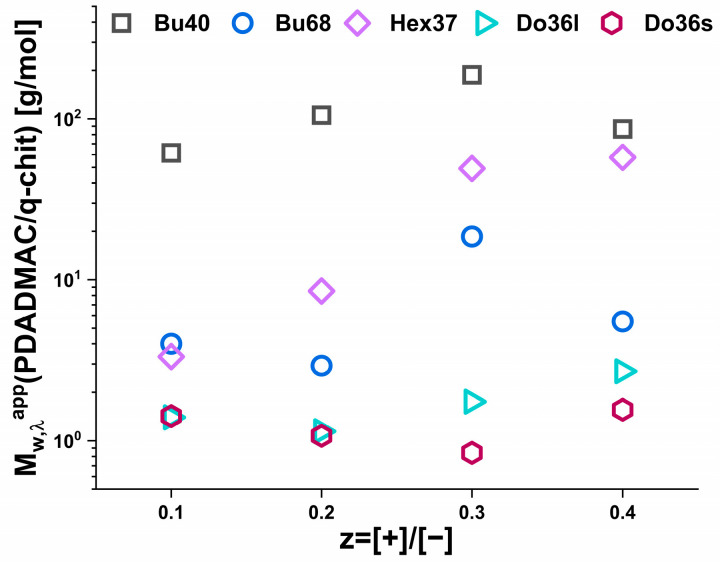
The ratio of apparent molecular weight ($M_{w,\lambda }^{app}$) of the IPECs obtained by complexing solutions of 5 g/L AlkA-b-NaPa with different amounts of polycation from turbidity measurements (at 632.8 nm).

Compared to the simple prediction for the masses by the addition of polycation and its complexation onto existing aggregates (see Figure S1), the increase was always much more pronounced for the addition of PDADMAC, while for q-chit this simple model often described the situation well, especially for smaller values of z. It was also very interesting to look at the ratio of the $M_{w,\lambda }^{app}$ values obtained for the same z value upon complexation with PDADMAC or q-chit, as shown in Figure 3. For the dodecyl polyacrylates, only somewhat larger values were observed for PDADMAC, while for Hex37 and Bu68, the increase by a factor of 3 to 50 generally became larger with increasing z. For Bu40, this factor was almost constant by about 100. Apparently, the size increase seen in turbidity depended strongly on the type of hydrophobic polyanion.

### 3.3. Static Light Scattering (SLS)

In order to confirm the structural information obtained by turbidity regarding the size of the IPEC aggregates formed upon admixing either PDADMAC or q-chitosan to the anionic copolymer micelles, we performed comprehensive light scattering experiments in which the mixing ratio z = [+]/[−] was increased systematically up to a value of z = 0.4. At this value, all samples were visually homogeneous, whereas higher values (higher than z = 0.4) of precipitation or coacervation were observed for all systems.

**Figure 4:**
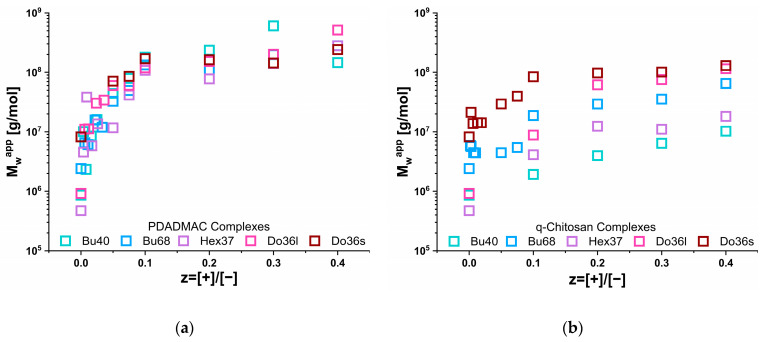
The apparent molecular weight (M_w_^app^) of the IPECs obtained by complexing solutions of 5 g/L AlkA-b-NaPa with different amounts of polycation (**a**) PDADMAC or (**b**) q-Chitosan via SLS.

Looking at the pure polyanions, one could observe the much weaker aggregation of the butyl- and hexylacylate copolymers compared to the dodecylacrylate, which is in good agreement with previous studies on the self-assembly of the pure copolymers [28]. The apparent molecular weight (M_w_^app^) as a function of z is shown in Figure 4 and confirms the substantially different behaviour for the IPECs formed with q-chit (Mw = 180 kDa) and PDADMAC (Mw = 150 kDa), as already seen in the turbidity measurements (Figure 2, a direct comparison between the results of turbidity and static light scattering results is shown in Figure S1). For q-chit, a continuous increase (or rather constant value for Do36s or Hex37) was observed, except for the addition of a polyelectrolyte onto an existing micellar copolymer aggregate. When comparing the increase with the one expected simply from assuming the stoichiometric addition of polycation to the existing structure of the anionic copolymer (Figure S1), one observed that this picture was quite well fulfilled for the complexes with q-chit. In contrast, PDADMAC addition was only in good agreement for the Do36s, while for all other copolymers, a much more marked increase was seen that demonstrates the formation of much larger complexes. As seen in the turbidity data, for the addition of PDADMAC, there was a drastic increase in scattering intensity at low z values that in light scattering was even more pronouncedly visible already for the addition of very small amounts.

This is a very intriguing difference that could indicate that the q-chit simply binds to the surface of the charged micellar aggregates, while the PDADMAC binds only partially at a low z and instead bridges to other micellar aggregates, thereby leading to the formation of clusters of such aggregates, which on average are 5 to 100 times larger than individual copolymer micelles, explaining the higher M_w_^app^ observed for the PDADMAC complexes. Interestingly, this effect was similarly seen for Do36s and Do36l, which by themselves form well-defined copolymer micelles [28], as well as for the butyl acrylates that form alone only rather ill-defined micellar aggregates. Apparently, the presence of PDADMAC transforms all the different copolymers at higher z values into about the same-sized complexes. However, the relative increase in Mw was much more pronounced for the butyl acrylates, as they formed only rather small aggregates in the absence of polycation.

Interestingly, the q-chit appeared to lead to a similar bridging and cluster formation for Do36s but to a lower extent than PDADMAC. For the other AlkA-b-NaPa systems, this did not lead to such an effect at all but instead fostered their transformation into compacted aggregates, which were then surrounded by the corresponding IPEC shell. In general, this indicated a stronger binding of the q-chit to the acrylate, resulting in more compact structures.

Looking at the aggregation numbers of the polyanions, given in Figure S2, for PDADMAC, rather constant numbers of 5000–10,000 were found for z equal to 0.1 and higher for all the different polyanions studied. This also confirmed that the aggregates seen in light scattering were not simple complexes of a micellar type but, with this size, must be more likely clusters of such micellar aggregates. In contrast, for q-chit, these values increased from about 100 for Bu40 to 500 for Do36s. Apparently, here the size of the structures formed was strongly dependent on the type of polyanion, and correspondingly differentiated complex structures were formed.

**Figure 5:**
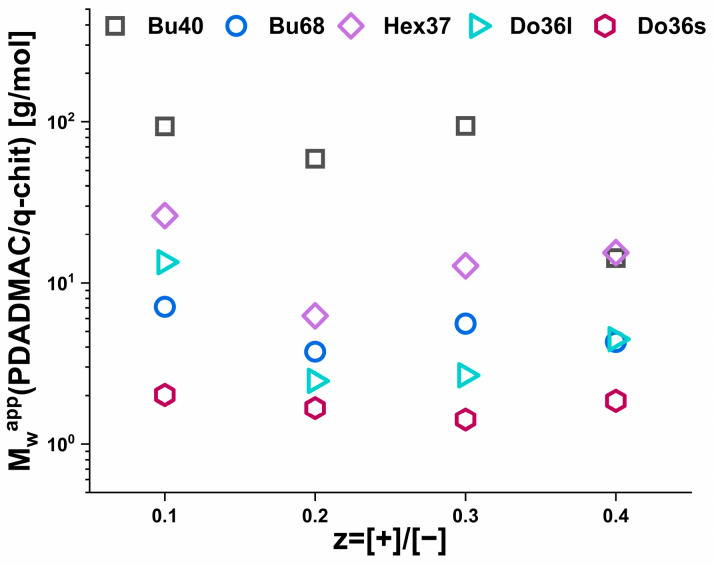
The ratio of apparent molecular weight (M_w_^app^) of the IPECs obtained by complexing solutions of 5 g/L AlkA-b-NaPa with different amounts of polycation from SLS experime.

The ratio of the apparent molecular weight of IPECs obtained through static light scattering experiments for the same z value using two different types of polycations—PDADMAC and q-chit was shown in Figure 5. Although the ratio decreased slightly from z = 0.1 to 0.2, it remained somewhat unchanged with further addition of polycation. This observation was consistent with the results obtained from turbidity measurements, indicating that the increase in size is dependent on the hydrophobicity of the polyanion used.

### 3.4. Small Angle Neutron Scattering (SANS)

**Figure 6:**
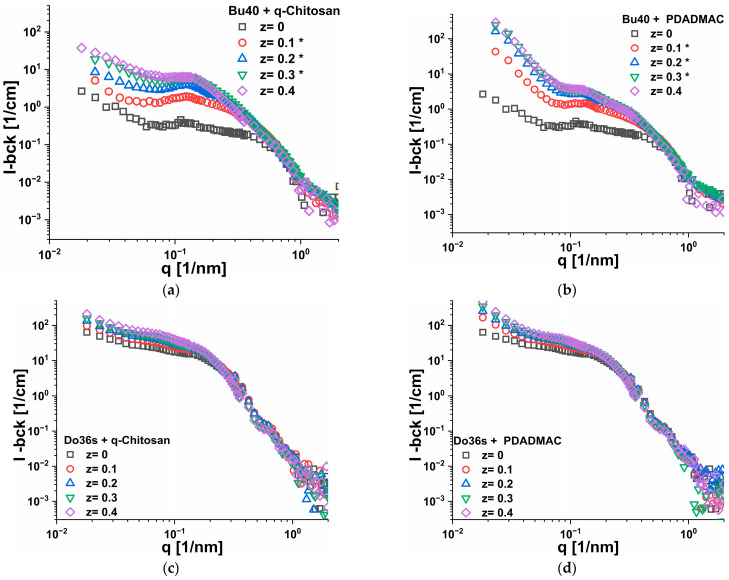
SANS intensity as a function of q for all z ratios from 0 to 0.4 for samples of 5 g/L (**a**,**b**) nBu_40_-b-AA_167_ (Bu40) or (**c**,**d**) nDo_36_-b-AA_71_ (Do36s) complexed with different amounts of either q-chitosan or PDADMAC (data from LLB which were corrected with respect to HZB data and ones from HZB which are marked as *. see Supplementary Materials Section S6.2).

To gain more detailed structural insights, SANS experiments were conducted on some selected copolymer micelles, Bu40 and Do36s, representing the cases of weak aggregation and formation of well-defined larger spherical micelles, respectively, that were complexed with either q-chit or PDADMAC. The obtained SANS intensity data as a function of q are shown in Figure 6, and they were in good agreement with the light scattering data with respect to the fact that at low q, higher scattering intensities were always observed for the addition of PDADMAC compared to that of q-chit, where this effect was much more pronounced for Bu40 compared to Do36s. Apparently, the smaller aggregates of the Bu40 could become more easily interconnected within a larger network by the addition of PDADMAC (Figure 6a,b). At the same time, they also became more compacted, as seen by the increase in the intermediate q-range of 0.1–0.2 nm^−1^. The slope at low q for the PDADMAC complexes with Bu40 is ~3.5 for the higher z values (see Figure S9), thereby indicating the formation of rather compacted structures larger than could be observed in the SANS experiments (which for the selected q-range means were at least larger than 100 nm), but which were covered in the light scattering experiments shown before.

The SANS data showed that for Do36s (Figure 6c,d), already without the addition of polycation, well-defined globular aggregates were present (some increasing toward lower q was seen, which was either due to some attractive interaction or larger aggregates). They become somewhat bigger by the addition of the polycation but apparently retain their globular structure. This effect was somewhat more pronounced for q-chit compared to PDADMAC, as expected from the fact that the mass per charge that became deposited in the IPEC shell was about twice for q-chit compared to PDADMAC. In addition, an increasing intensity at low q was seen with an increasing polycation addition, which was again more pronounced for PDADMAC, compared to the q-chit, which could be attributed to the formation of a polycation corona of the aggregates that were also interconnecting them.

The situation was very different for the Bu40, where a much more marked increase in intensity in the mid-q range around 0.1 nm^−1^ was seen for the addition of both polycations. This meant that here, initially, no larger self-assembled aggregate was present and substantial aggregation was induced (seen in the q-range of 0.08–0.4 nm^−1^) by the addition of the polycation, and much larger and more compacted aggregates were formed. The effect was generically similar for both polycations but more marked for q-chit.

This also led to the formation of a marked correlation peak, which was more pronounced for q-chit (Figure 6a) compared to adding PDADMAC (Figure 6b), as it was obscured by a low q increase. The overall scattering intensity around q ~0.1 nm^−1^ was much higher for the addition of q-chit, showing that, here, appear larger and more well-defined aggregates were formed than for the complexation with PDADMAC. Of course, in addition, it must be noticed that q-chit had a higher Mw per charged unit, and, accordingly, more scattering power was generated in the process of complexation. At first glance, this was in strong disagreement with the observations of static light scattering (Figure 4), but in SANS, we looked at a much smaller size range. However, the much higher SLS intensity seen for PDADMAC was reflected in the SANS curves in the large upturn of intensity in the low q-range, while for q-chit here, only some increase was seen. An explanation for such behaviour would be that PDADMAC leads to a much more pronounced interlinking of the different IPEC copolymer aggregates (here, one has to keep in mind that the size of these aggregates was ~5–10 nm, while the stretched lengths of the PDADMAC and the q-chit chains was ~460 nm and ~440 nm, respectively) (see Supplementary Materials Section S5). This more pronounced interlinking by PDADMAC was confirmed by the observation that the macroscopic viscosity of the IPEC solutions was higher by a factor of two for PDADMAC compared to the corresponding q-chit solutions (Table S4).

The peak positions were similar for complexation with q-chit and PDADMAC, which also indicated that the size of the formed complex aggregates was similar. However, the sharp increase at low q seen for PDADMAC showed that they were contained in much more interconnected clusters. In contrast, with q-chit, more isolated particles were formed, which still interacted repulsively for the case of Bu40 (correlation peak), while for the Do36s, a more marked core–shell structure appeared to be visible. SANS data of the complexes of PDADMAC with Bu40 were analysed in the low q range via a simple power law, which for z, showed a larger 0.1 scaling of I(q) ~ q^−3.5^, indicating that here one seemed to see rather well-defined larger cluster domains (See Figure S9). On the other hand, the q-chit complexes with Bu40 showed a power law of I(q) ~ q^−1.5^ for higher z, which indicated the formation of much more open structures (see Figure S8).

**Figure 7:**
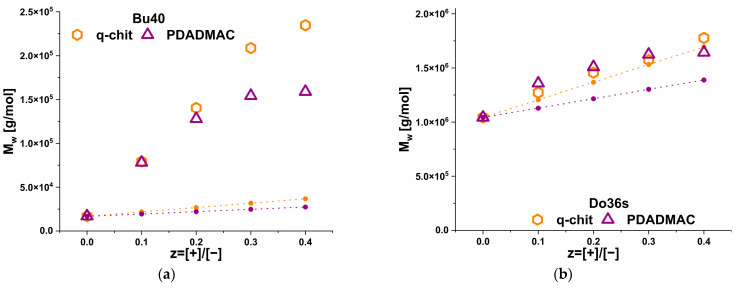
M_w_ of the primary aggregates calculated from I(0) as obtained from a Guinier fit in the mid-q range (for details see Supplementary Materials Section S6.3) as a function of z for the complexation of (**a**) nBu_40_-b-AA_167_ (Bu40) and (**b**) nDo_36_-b-AA_71_ (Do36s) with q-chit and PDADMAC. Theoretical predictions of the Mw of the complexes for simple addition on the structures existing at z = 0 as a function of z are shown as dotted lines.

In order to quantify the scattering behaviour further, the mid-range indicated the presence of globular structures on a scale of 5–20 nm; SANS data of the complexes was fitted to the shape-independent Guinier law, thereby obtaining I(0) and the radius of gyration R_g_. This was conducted in the q-range, which can be found in Figures S4–S7. From I(0), the Mw of these aggregate structures was calculated according to Equation (S3), and the obtained values are given in Figure 7 and Table 2.

For Do36s, R_g_ increased slightly from 8.0 nm for the pure micelle to less than 11 nm for z = 0.4. This meant that, here, we saw a systematic but relatively small increase that was in good agreement with the evolution of the M_w_ data shown in Figure 7. On the other hand, as seen in Table 2, the R_g_ of the pure Bu40 system was 3.2 nm, and upon the formation of aggregates by adding polycation, it increased up to 8.9 nm. For the M_w_ values, one found that for Do36s, they increased for both polycations by about 30% from 1 × 10^6^ g/mol to 1.3⸱10^6^ g/mol. For Bu40, the situation was quite different. An addition of polycation into nBu_40_-b-AA_167_ micelles resulted in an increase by a factor of 6 from 1.4 × 10^4^ g/mol to ~8 × 10^4^ g/mol. Q-Chitosan complexes were slightly larger than PDADMAC complexes, and the molecular weight of the complexes increased with an increase in the charge ratio. From the M_w_ values, one could straightforwardly calculate the aggregation numbers N_agg_, assuming all polymers could be aggregated in these aggregates (See Supplementary Materials Section S6.3, Equations (S5)–(S7)). This shows (Table 2) that the Bu40 N_agg_ was always in the range of 3–6, while the polycation was contained in a number of 0.1–0.7 (See Table 2), thereby explaining that these complexes necessarily had to be bridged by polycation. For the Do36s, the situation was much different. Here, N_agg_ for the Do36s was rather constant around ~85, and N_agg_ of the polycation increased systematically from around 0.9 to 3.8 (See Table 2). This explains why rather compact and slightly interconnected aggregates were formed. As shown in Figure 7, for q-chit added to Do36s, the M_w_ values followed very nicely the theoretical prediction for simply adding the polycation onto the existing anionic copolymer micelles, while for PDADMAC the increase was somewhat larger. In contrast, for Bu40, a very substantial increase in M_w_ was seen that demonstrated that here the formation of compacted aggregates was largely driven by the presence of the polycation. This was further quantified by the rather low aggregation numbers given in Table 2.

An effective density, $\rho_{eff}$, calculated from Equation (S9), could also quantify the IPEC’s compactness. The effective densities of the IPECs based on Bu40 were much lower than that of Do36s complexes. In general, q-chit complexes achieved higher effective densities in comparison to PDADMAC complexes. This confirmed again the higher compactness of the complexes with q-chit than PDADMAC. Moreover, considering an increase in z ratio, the concentration of polycation resulted in a decrease in the effective density of the IPECs.

As a next step of quantitative analysis, we used the position of the correlation peak (q_max_) seen for the nBu_40_-b-AA_167_ samples, as that which should give the mean spacing *d* (=2π/q_max_) between the aggregates. Assuming all of the copolymer to be aggregated and all of the added polycation to be bound in a more or less compact IPEC shell (and assuming the homogeneous distribution of the complexes in space), we could proceed to calculate the volume fraction Φ of dispersed aggregates, for which we assumed a density ρ of 1.1 g/mL. Further assuming that the mean spacing d could be approximated by placing the aggregates on a primitive cubic lattice, we calculated their aggregation number, the radius of the core *R* and the molecular weight *M_w_* as:

(10) $$R=\sqrt[{3}]{\frac{3*\Phi *d^{3}}{4\pi }}$$

(11) $$M_{w}=N_{Av}*\rho *\left(\frac{4\pi R^{3}}{3}\right)$$

The obtained values are summarized in Table 3, and the values for *M_w_* were generally rather constant in the range of 5–6·10^5^ g/mol and thereby generically similar to the ones obtained from the intensity (Table 2) but varying less as a function of z and polymer. However, here it has to be pointed out that the values in Table 2, arising from the absolute intensity, were more reliable, as for the values in Table 3, a homogeneous distribution of the aggregates in space was assumed, which especially for the Bu40 might not have been the case, as seen by the intensity upturn at a low q which indicated clustering. In addition, we saw much higher aggregation numbers in Table 3 (compared to the ones given in Table 2), which indicated that for the not-so-hydrophobic Bu40 copolymer, only a fraction of the molecules was really contained in these compacted aggregates and a larger part of it was contained in a less compacted form.

## 4. Discussion

We studied the complexation of anionic copolymer aggregates with alkyl acrylates as the hydrophobic part and polyacrylates as the hydrophilic part. The hydrophobicity of the hydrophobic block was varied using alkyl chains from butyl to dodecyl, and also the length of the hydrophilic block was varied. The dodecyl polymer was sufficiently hydrophobic to form spherical micelles with an aggregation number of about 40–80, while the aggregates became smaller and less defined with the decreasing length of the alkyl chain of the acrylates [28]. These anionic copolymer aggregates were complexed by the polycations PDADMAC or q-chitosan, leading to aggregates with a hydrophobic core of the alkyl acrylate surrounded by an IPEC shell of PAA and PDADMAC or q-chit, which were stabilised in an aqueous solution by the remaining excess PAA chains. Especially for the butyl acrylates, which show by themselves as having a rather weak aggregation tendency, the addition of polycation induced a marked increase in aggregation on a local scale of ~5–8 nm, as well as the formation of larger structures, as seen by light scattering.

**Figure 8:**
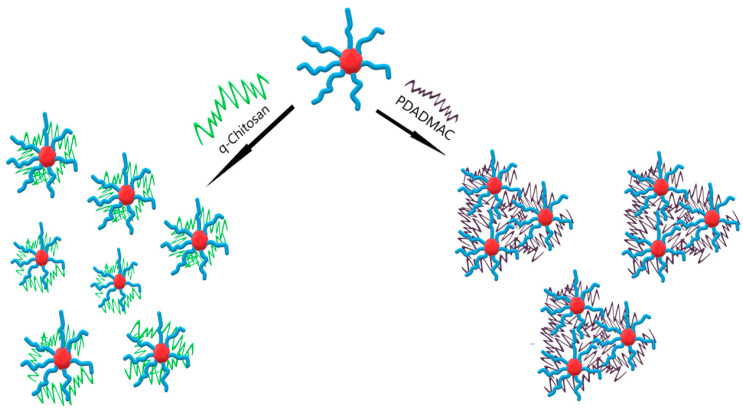
Sketch of formed IPECs either from q-chit or PDADMAC. Red colour represents the hydrophobic core of the polyanion, while blue colour shows the hydrophilic block of the polyanion. q-chit is shown in green, while dark purple is the colour of PDADMAC.

Similar-sized aggregates were formed upon complexation with PDADMAC or q-chit; for PDADMAC, light scattering and the low q-range of the SANS data showed a very marked increase in intensity, which could be interpreted as an interconnection of the different IPEC aggregates by the polycation. This was not surprising since the polycations employed for complexation were much longer (~450 nm) than the average spacing between the IPEC aggregates, and SANS showed that, typically, there was less than one polycation contained in an aggregate. However, despite the fact that q-chit and PDADMAC had a similar stretched length, they showed here markedly different behaviour. This may be attributed to the fact that q-chit had an intrinsically lower affinity to be dissolved in water and bound more strongly to the polyacrylate. As a result, it formed a more compacted IPEC shell, while PDADMAC could extend more easily out from the shell of the IPEC micelles into the aqueous surroundings, thereby being able to bridge to neighbouring aggregates. This different structural behaviour is depicted in Figure 8 and was confirmed by the macroscopic viscosity of the solutions of the different complexes, which was more than twice as high for PDADMAC complexes compared to those with q-chit (Table S4).

## 5. Conclusions

Our experiments on IPEC formation with amphiphilic block copolymers with a variable extent of hydrophobicity of the hydrophobic block demonstrate how sensitive IPEC structures react to the parameter hydrophobic modification. Accordingly, this is an important parameter in the design of IPEC systems. However, the molecular details of the complexing homopolyelectrolyte are also important, as seen in our experiments in the different behaviour of PDADMAC and quaternized-chitosan, where at the same polymer length, the PDADMAC led to bridging and cluster formation and the q-chit not. Correspondingly, the choice of the complexing polyelectrolyte offered a facile way to control the structural properties of such micellar IPECs and thereby of their properties as they could be interested in using them as tailor-made ionic assemblies, for instance, for delivery purposes.

## Figures and Tables

**Table 1:** The experimentally determined chemical formula of the produced polymers, abbreviation of the name of the polymers for this study, the number average molecular weight (M_n_) of hydrolysed polymer and polydispersity index (Đ) from GPC experiments.

Chemical Formula	Abbreviation	M_n_ [g/mol]	Đ
nBu40-b-AA167	Bu40	17,180	1.15
nBu68-b-AA167	Bu68	20,680	1.19
nHex37-b-AA169	Hex37	17,960	1.12
nDo36-b-AA127	Do36l	17,860	1.20
nDo36-b-AA71	Do36s	13,950	1.28

**Table 2:** Radius of gyration R_g_, aggregation number for polyanion and polycation Nagg [−] and Nagg [+], respectively, and effective density $\rho_{eff}$ at a different charge ratio z, as determined from a Guinier approximation of the data from SANS experiments. (See Supplementary Materials Section S6.3 for details of the calculation).

Bu40 Complexes	z = [+]/[−]	Rg [nm]	Nagg [−]	Nagg [+]	$\;\rho_{eff}\;[g/\mathrm{mL}]$
	0	3.3	1	0	0.19
with q-chit	0.1	5.4	3.6	0.1	0.20
	0.2	6.6	5.1	0.3	0.19
	0.3	7.5	6.4	0.5	0.20
	0.4	8.9	6.3	0.7	0.13
with PDADMAC	0.1	6.4	3.9	0.1	0.12
	0.2	7.1	5.7	0.2	0.14
	0.3	7.7	6.1	0.3	0.13
	0.4	8.8	5.7	0.4	0.09
**Do36s Complexes**	**z = [+]/[−]**	**Rg [nm]**	**Nagg [−]**	**Nagg [+]**	$\;\rho_{eff}\;[g/\mathrm{mL}]$
	0	8.0	74.6	0	0.81
with q-chit	0.1	8.5	78.8	0.9	0.82
	0.2	9.5	79.4	1.9	0.67
	0.3	10.1	76.6	2.8	0.61
	0.4	10.7	78.3	3.8	0.58
with PDADMAC	0.1	8.8	89.8	0.7	0.79
	0.2	9.4	92.6	1.4	0.72
	0.3	9.9	93.1	2.2	0.66
	0.4	10.3	88.3	2.7	0.60

**Table 3:** The radius R, molecular weight Mw and aggregation number for polyanion Nagg [−] calculated from the correlation peak position of the complexes formed in solutions of 5 g/L of Bu40 upon the addition of different amounts of q-Chitosan or PDADMAC.

Bu40 Complexes	z = [+]/[−]	R [nm]	Mw (⸱10^5^) [g/mol]	Nagg [−]
with q-chit	0.1	5.82	5.46	24.5
	0.2	6.18	6.54	23.9
	0.3	6.18	6.56	20.2
	0.4	5.77	5.34	14.2
with PDADMAC	0.1	5.56	4.76	23.9
	0.2	5.80	5.40	23.9
	0.3	5.65	5.00	19.8
	0.4	5.71	5.16	18.5

## Data Availability

The data presented in this study are available on request from the corresponding authors.

## Supplementary Materials

The following supporting information can be downloaded at: https://www.mdpi.com/article/10.3390/polym15092204/s1, Table S1: Zeta Potential of the complexes of Bu40 and Do36s with q-chitosan and PDADMAC at different charge ratio z. Table S2: Refractive index increment dn/dc of the different polymers at 25 °C measured at 620 nm. Table S3: SLD of the complexes. Table S4. Viscosity measurements of Bu40 Complexes. Figure S1: Direct comparison of the $M_{w}^{app}$ values for the different copolymers upon complexation with PDADMAC (left) or q-chit (right) obtained from the turbidity and the static light scattering measurements. The theoretical Molecular Weight values are shown as UV_th and SLS_th. The theoretical Mw values were calculated with the assumption that the micelle size remains constant and the corresponding amount of polycation is simply complexing these micelles. Figure S2: The aggregation number of polyanion of the IPECs obtained by complexing solutions of AlkA-b-NaPa with different amounts of polycation (a) q-Chitosan or (b) PDADMAC via SLS. Figure S3: The measured TTAB reference sample at the facilities HZB (dark grey), LLB (red) and the corrected LLB data (blue). Figure S4: The Guinier approximation plots for Bu40 Complexes with q-Chitosan at different charge ratios z (the fitted q range ~0.01–0.03 Å^−1^). Figure S5: The Guinier approximation plots for Bu40 Complexes with PDADMAC at different charge ratios z (the fitted q range ~0.01–0.02 Å^−1^). Figure S6: The Guinier approximation plots for Do36s Complexes with q-Chitosan at different charge ratios z (the fitted q range ~0.009–0.03 Å^−1^). Figure S7: The Guinier approximation plots for Do36s Complexes with PDADMAC at different charge ratios z (the fitted q range ~0.009–0.03 Å^−1^). Figure S8: The Power Law plots for Bu40 Complexes with q-Chitosan at different charge ratios z. Figure S9: The Power Law plots for Bu40 Complexes with PDADMAC at different charge ratios z [37,38].
