# Supplementary material for: A Comparison of Interpolyelectrolyte Complexes (IPECs) Made from Anionic Block Copolymer Micelles and PDADMAC or q-Chitosan as Polycation
Source: Polymers (Basel). 2023 May 6;15(9):2204. doi: 10.3390/polym15092204 (PMC10181488; doi:10.3390/polym15092204)
Supplement: Supplementary file 1 [file polymers-15-02204-s001.zip › polymers-2329749-supplementary.pdf]

A Comparison of Interpolyelectrolyte Complexes (IPECs) Made from Anionic Block Copolymer Micelles and PDADMAC or q-Chitosan as Polycation

Özge Azeri[1]*, Dennis Schönfeld[1], Bin Dai[1], Uwe Keiderling[2], Laurence Noirez[3], Michael Gradzielski[1]*

1: Stranski-Laboratorium für Physikalische und Theoretische Chemie, Institut für Chemie, Technische Universität Berlin, D-10623 Berlin, Germany

2: Helmholtz-Zentrum Berlin (HZB) für Materialien und Energie GmbH, Berlin, Germany

3: Laboratoire León Brillouin CEA-CNRS, Université Paris-Saclay, F-91191 Gif-sur-Yvette Cedex, France

Supplementary Information

1. Zeta-potential

Table S1. Zeta Potential of the complexes of Bu40 and Do36s with q-chitosan and PDADMAC at different charge ratio z.

	**Zeta Potential, ζ [mV]**			
	**q-Chitosan Complexes**		**PDADMAC Complexes**	
**z= [+]/[-]**	**Bu40**	**Do36s**	**Bu40**	**Do36s**
**z = 0**	-38.5	-35.4	-38.5	-35.4
**z = 0.2**	-39.9	-34.8	-39.4	-35.9
**z = 0.4**	-24.8	-29.7	-35.7	-31.1

2. MW of the Complexes from Turbidity (UV) and SLS experiments

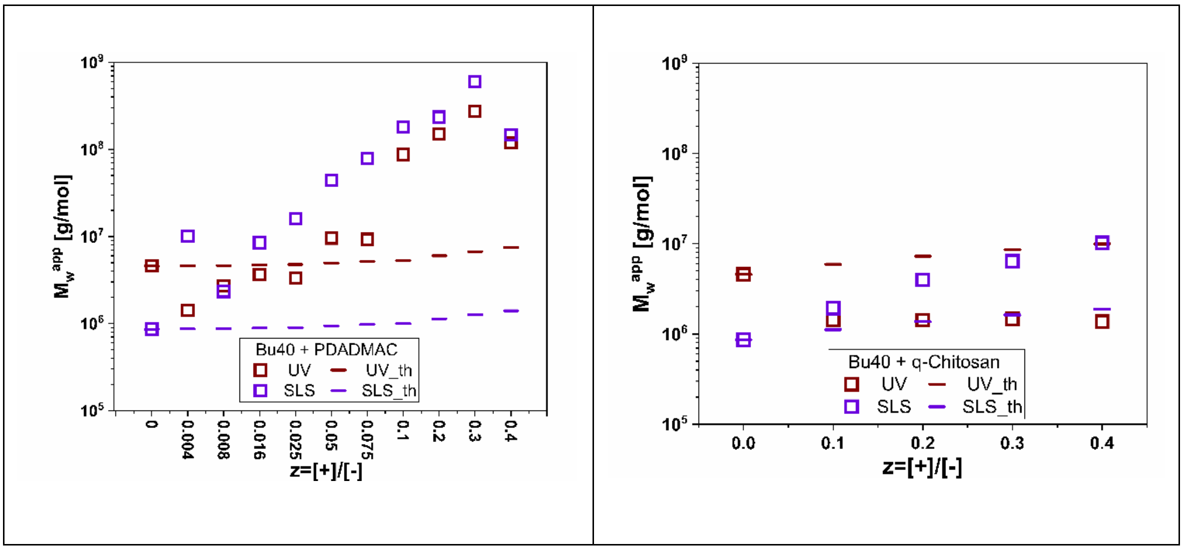

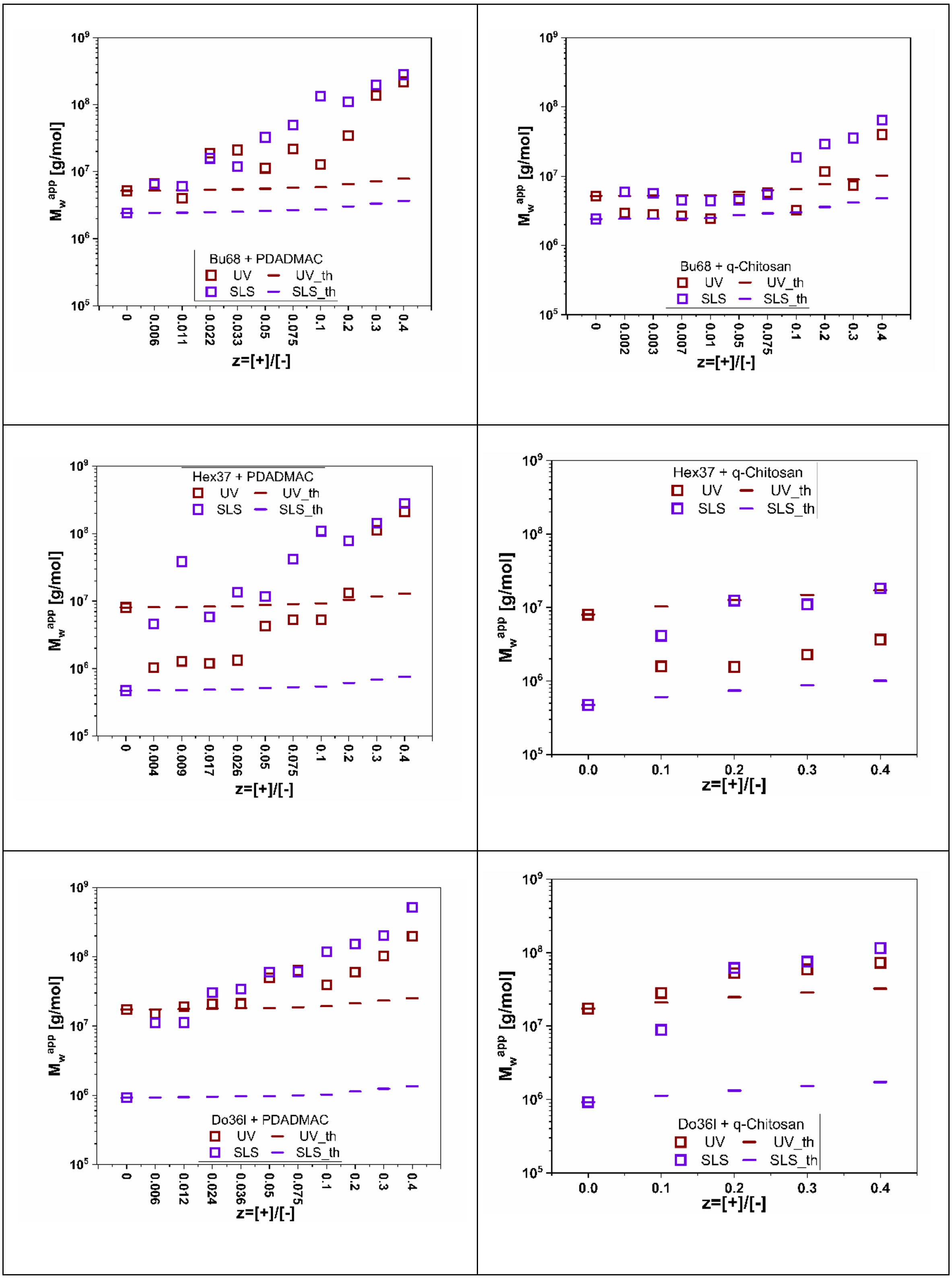
Bu68 + PDADMAC
UV
UV_th
SLS
SLS_th
M_w^{app} [g/mol]
z=[+]/[-]
Bu68 + q-Chitosan
Hex37 + PDADMAC
Hex37 + q-Chitosan
Do36I + PDADMAC
Do36I + q-Chitosan

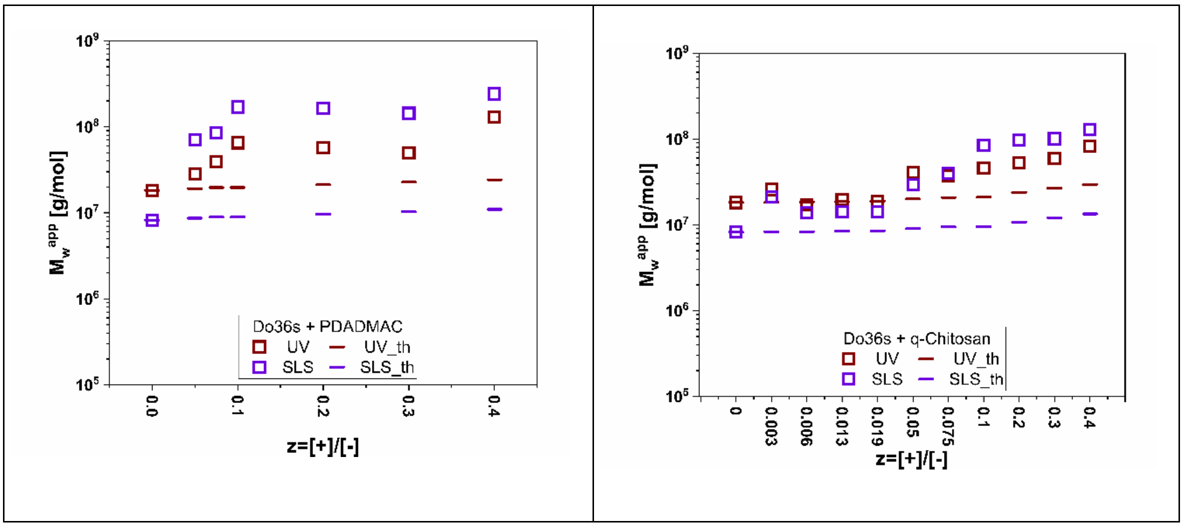

Figure S1. Direct comparison of the M_w^{app} values for the different copolymers upon complexation with PDADMAC (left) or q-chit (right) obtained from the turbidity and the static light scattering measurements. The theoretical Molecular Weight values are show as UV_th and SLS_th. The theoretical Mw values were calculated with the assumption that the micelle size remains constant and the corresponding amount of polycation is simply complexing these micelles.

3. Static Light Scattering

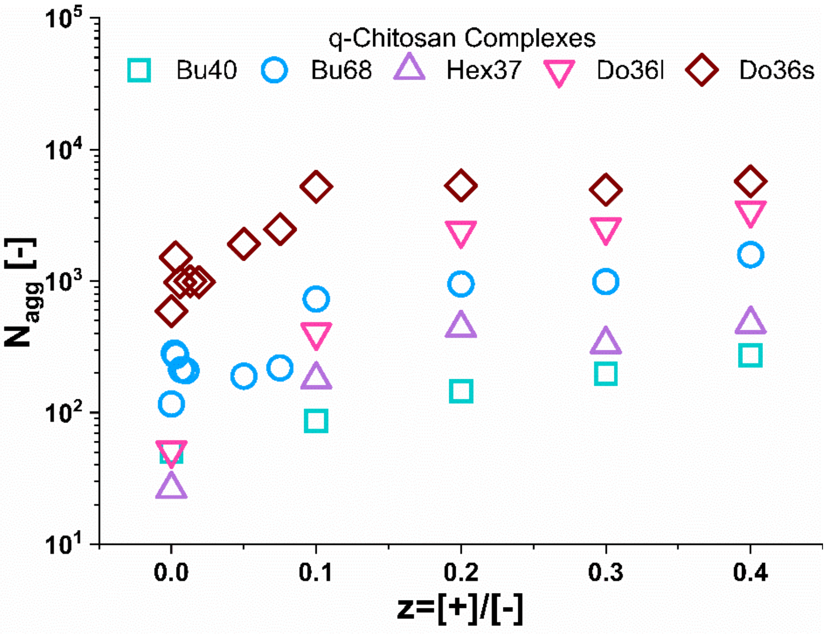

(a)

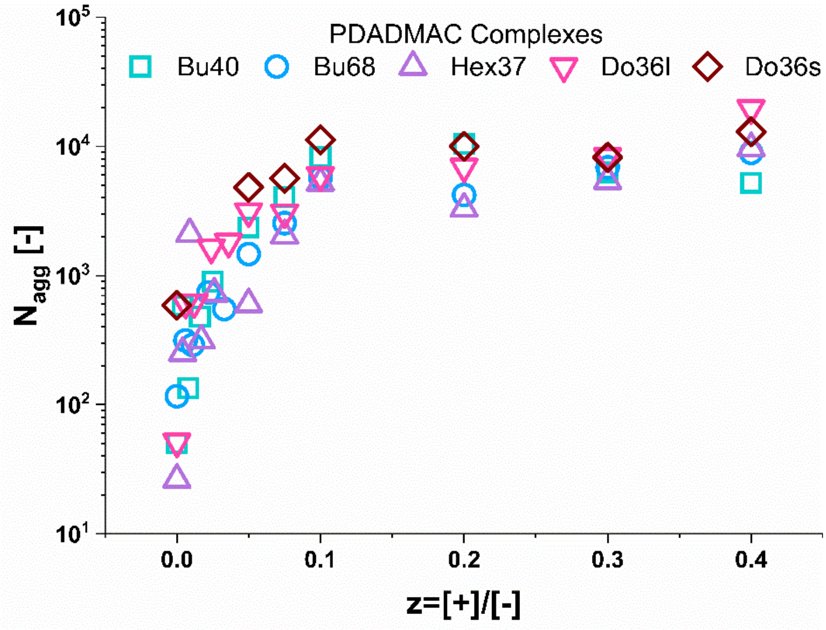

(b)

Figure S2. The aggregation number of polyanion of the IPECs obtained by complexing solutions of AlkA-b-NaPa with different amounts of polycation (a) q-Chitosan or (b) PDADMAC via SLS.

4. Refractive index increment dn/dc

dn/dc of the complexes were calculated via

$$\left(\frac{d_n}{d_c}\right)_{IPEC} = \sum_i \left(\left(\frac{d_n}{d_c}\right)_i * \phi_i\right) \qquad \text{(S1)}$$

ϕ_i volume fraction of Polyanion or Polyanion in corresponding complex.

Table S2. Refractive index increment dn/dc of the different polymers at 25 °C measured at 620 nm.

**Polymer**	**dn/dc [mL/g]**
Bu40	0.149
Bu68	0.154
Hex37	0.158
Do36l	0.165
Do36s	0.158
q-Chitosan [1]	0.214
PDADMAC [2]	0.213

5. The Stretched Length of the Polycations

The contour length of PDADMAC is calculated as

$$L = \frac{M_w * l}{M_{w,mon}} = \frac{150000\,\frac{\mathrm{g}}{\mathrm{mol}} \; * \; 0.50\ \mathrm{nm}}{161.67\ \mathrm{g/mol}} = 464\ nm$$

Where $M_{w,mon}$ and l are the DADMAC monomer molecular weight and a unit length, respectively and M_w is the molecular weight of PDADMAC.

The stretched length of Chitosan is calculated as

$$L = \frac{M_w * l}{M_{w,ring}} = \frac{130000\ \mathrm{g/mol} \; * \; 0.57\ \mathrm{nm}}{168.9\ \mathrm{g/mol}} = 439\ nm$$

Where $M_{w,ring}$ and l are the molecular weight and the length of the averaged sugar ring of the Chitosan, respectively and M_w is the molecular weight of the original purified Chitosan.

6. Small Angle Neutron Scattering

6.1. SLD of the Complexes

SLDs of the complexes (SLD_{comp}) were calculated from the sum of the volume fractions Φ_i of the polyanion or the polycation multiplied with the SLD of the corresponding part (i)

$$SLD_{comp} = \sum_i (\phi_i * SLD_i) \tag{S2}$$

The assumption of 1:1 IPECs is the hydrophobic core added on the polycation and the hydrophilic chain of polyanion with respect to the amount of added polycation.

Table S3. SLD of the complexes

Complexes		z=[+]/[-]	SLD_{comp} [Å^{-2}]
**Bu40**	**PDADMAC**	0.1	1.192E-06
		0.2	1.110E-06
		0.3	1.045E-06
		0.4	9.928E-07
**Do36s**		0.1	7.375E-07
		0.2	7.208E-07
		0.3	7.063E-07
		0.4	6.936E-07
**Bu40**	**q-Chitosan**	0.1	1.302E-06
		0.2	1.303E-06
		0.3	1.304E-06
		0.4	1.304E-06
**Do36s**		0.1	8.315E-07
		0.2	8.882E-07
		0.3	9.329E-07
		0.4	9.690E-07

6.2. The correction of the SANS data

The correction was done with respect to the differences between reference TTAB measurement.

$$Imax\_HZB / Imax\_LLB = I\_shiftfactor = 1.408462$$

$$q_{max}\_HZB(at\ I_{max}) / q_{max}\_LLB(at\ I_{max}) = q\_\mathit{shiftfactor} = 0.927534$$

All LLB data was multiplied with the shift factor correspondingly and after that the corrected data was used for the characterization of complexes. The data from HZB was shown with * in Figure 6.

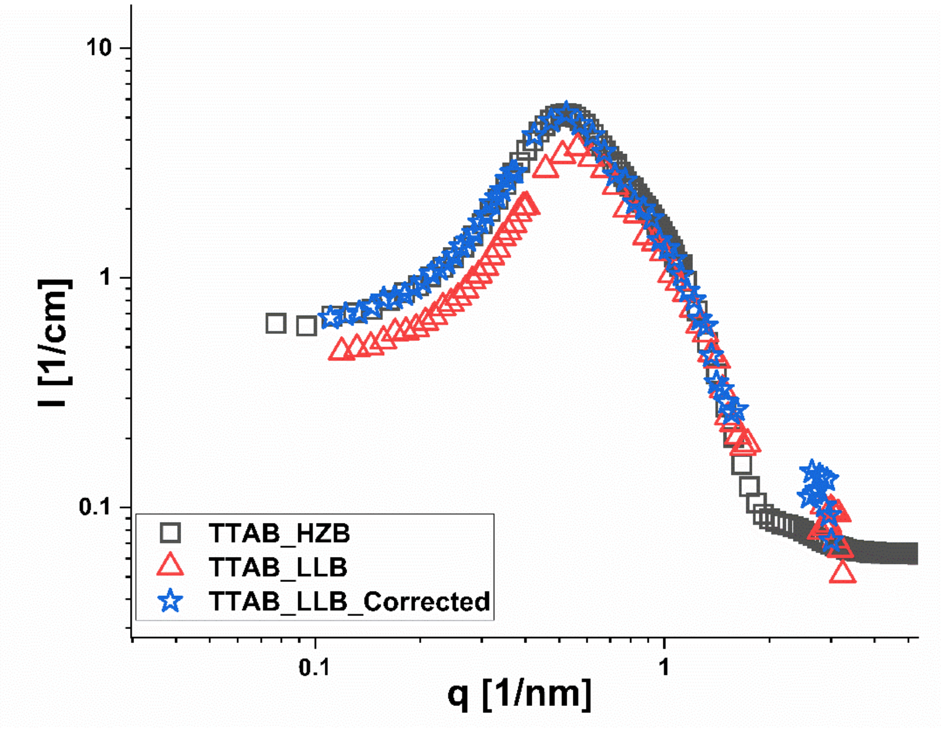

Figure S3. The measured TTAB reference sample at the facilities HZB (dark grey), LLB (red) and the corrected LLB data (blue).

6.3. Model Free Analysis- Guinier Approximation

Model-free analysis of SANS curves, yielding the extrapolated intensity by Guinier approximation at zero-scattering angle, I(0), the resulting apparent molecular weight M_w calculated via

$$M_w = \frac{I(0) * d^2 * N_{av}}{c * \Delta SLD^2} \qquad (S3)$$

where c is the concentration which was the sum of the added polycation with respect to desired z ratio onto the constant 5 g/L polyanion aggregates.

Molecular weight of Polyanion (Mw_PA) and Polycation (Mw_PC) can be found in 2.1. Materials Part and Table 1 in the main text.

f[-] and f[+] are the mol fraction of whole Polyanion and Polycation in complexes. Molecular weight of the complexes was calculated as follows.

$$\text{Mw\_comp} = \text{f}[-]\ \text{Mw\_PA} + \text{f}[+]\ \text{Mw\_PC} \qquad (S4)$$

Aggregation number of the complexes (Nagg_comp) are calculated from Mw of the complex (from Equation S3) and the Mw_comp (from Equation S4)

$$N_{agg} = \frac{M_W}{M_{W\_comp}} \tag{S5}$$

Aggregation number of corresponding Polyanion (Nagg [−]) and Polycation (Nagg [+]) in complexes is calculated

$$\text{Nagg}\ [-] = \text{f}[-]\ \text{Nagg\_comp} \tag{S6}$$

$$\text{Nagg}\ [+] = \text{f}[+]\ \text{Nagg\_comp} \tag{S7}$$

Assuming the aggregates as spherical, volume of the aggregates(V) with a radius of gyration (R_g)

$$V = \frac{4}{3}\pi {R_g}^3 \tag{S8}$$

The effective density of the complexes (ρ_{eff}) can be calculated with the following equation S9

$$\rho_{eff} = \frac{M_W}{V * N_{av}} \tag{S9}$$

where N_{av} is the Avogadro number.

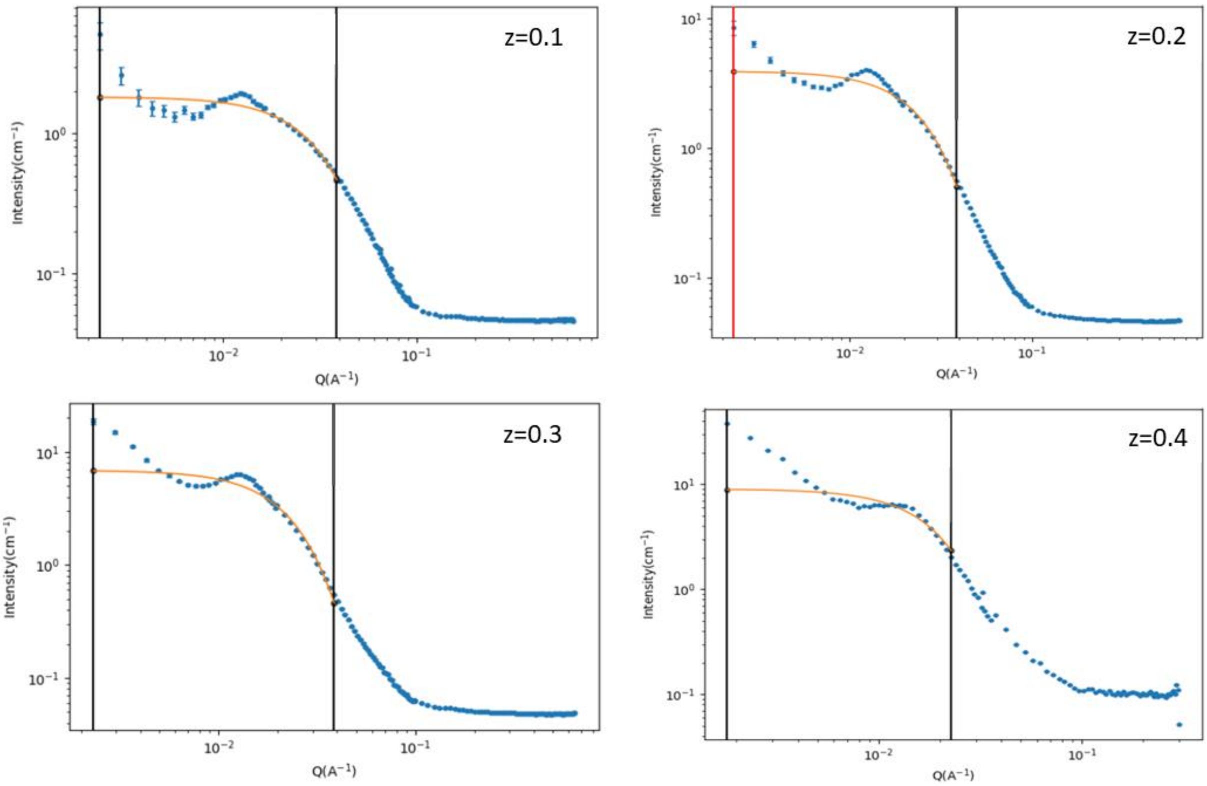

Figure S4. The Guinier approximation plots for Bu40 Complexes with q-Chitosan at different charge ratios z (the fitted q range ~0.01-0.03 Å^{-1}).

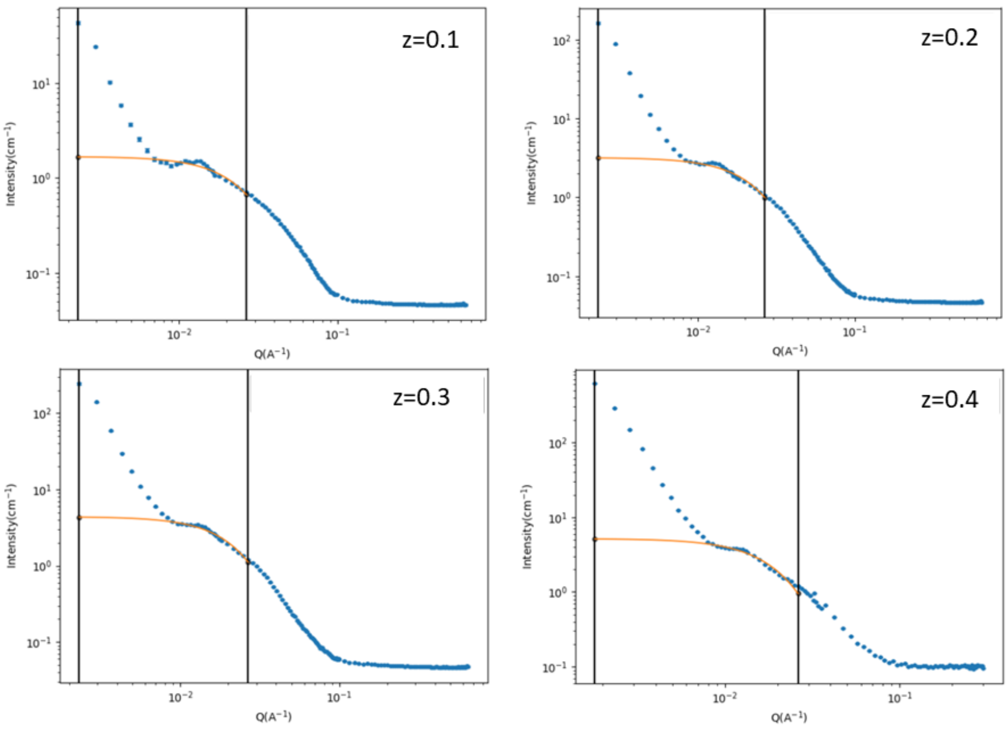

Figure S5. The Guinier approximation plots for Bu40 Complexes with PDADMAC at different charge ratios z (the fitted q range ~0.01-0.02 Å^{-1}).

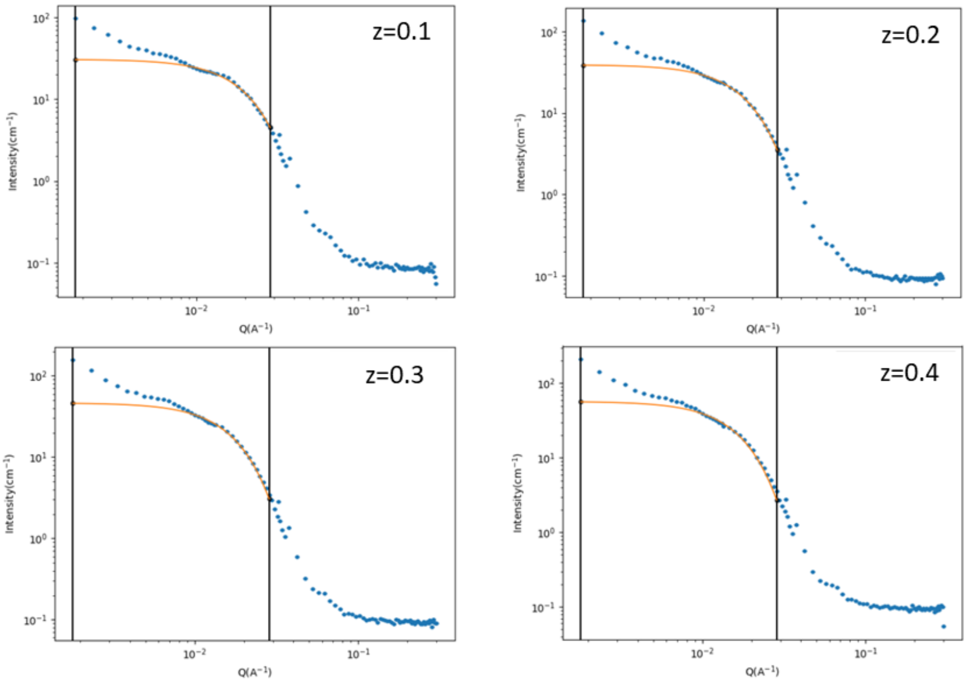

Figure S6. The Guinier approximation plots for Do36s Complexes with q-Chitosan at different charge ratios z (the fitted q range ~0.009-0.03 Å^{-1}).

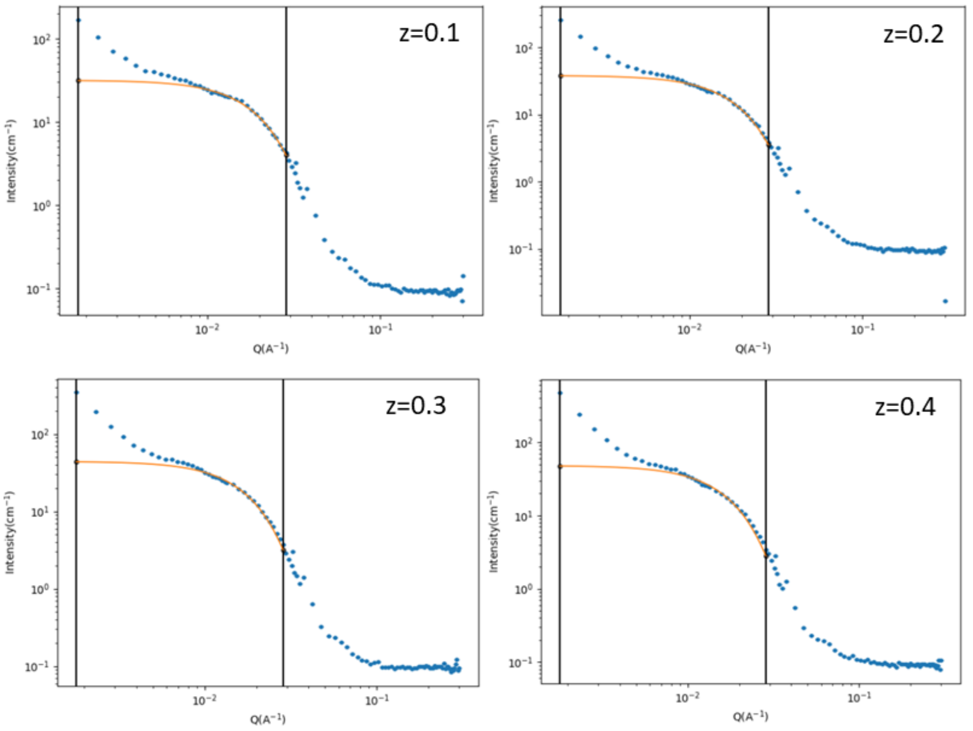

Figure S7. The Guinier approximation plots for Do36s Complexes with PDADMAC at different charge ratios z (the fitted q range ~0.009-0.03 Å^{-1}).

6.4. Slope at lower q for the Bu40 Complexes - The Power Law

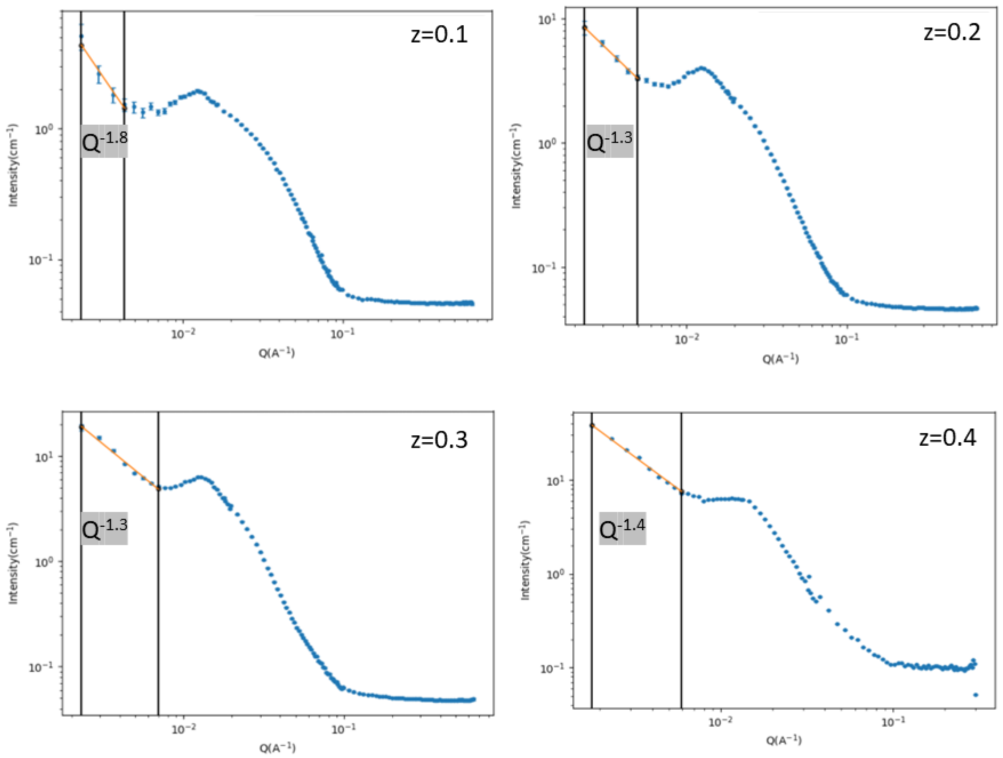

Figure S8. The Power Law plots for Bu40 Complexes with q-Chitosan at different charge ratios z.

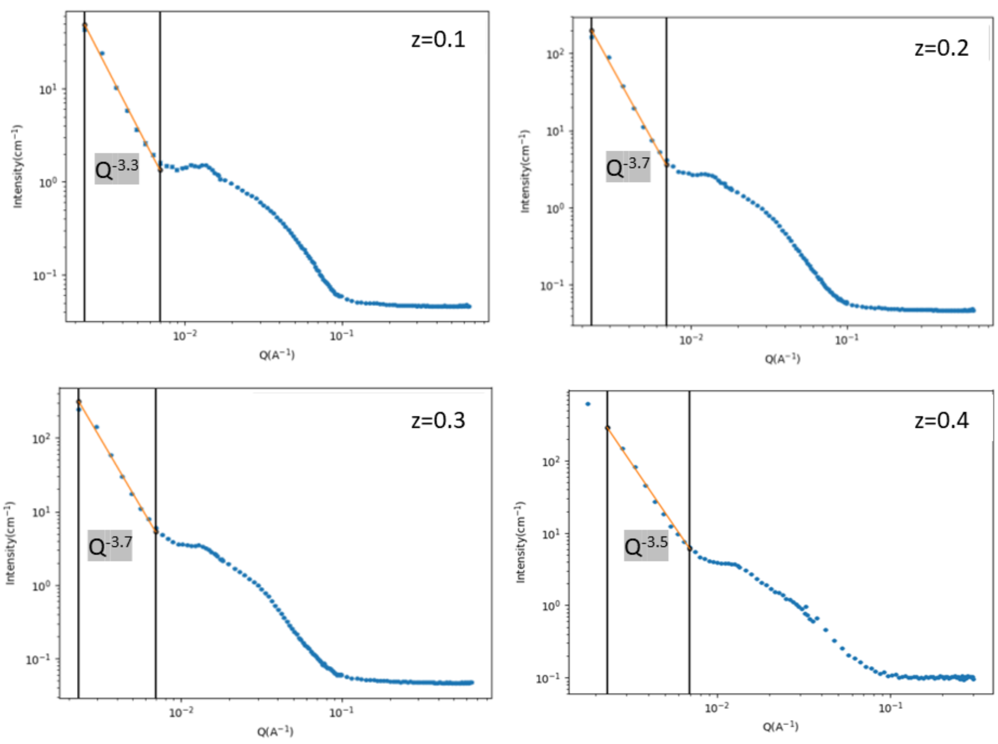

Figure S9. The Power Law plots for Bu40 Complexes with PDADMAC at different charge ratios z [1,2].

7. Viscosity measurements of Bu40 Complexes

Table S4. Viscosity measurements of Bu40 Complexes.

Bu40 Complexes	z=[+]/[-]	ν [mm²/s]	η [mPas]
**with q-Chit**	0.2	1.55	1.72
	0.4	1.50	1.67
**with PDADMAC**	0.2	3.18	3.52
	0.4	3.15	3.49

8. Some pictures of the obtained solutions

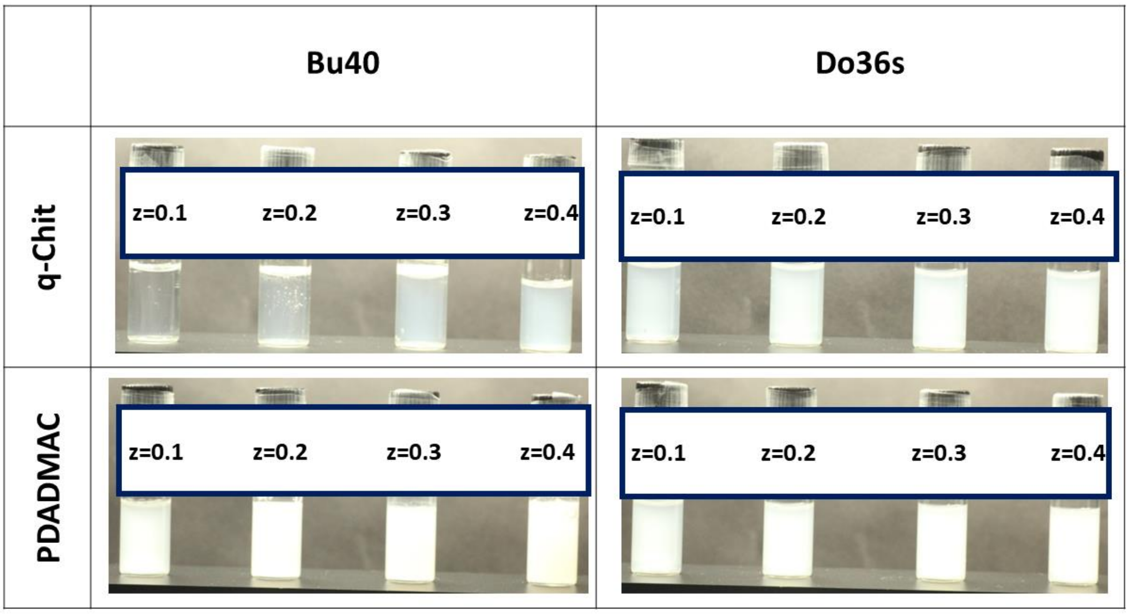

References

1. Chiappisi, L.; Prévost, S.; Grillo, I.; Gradzielski, M. Chitosan/Alkylethoxy Carboxylates: A Surprising Variety of Structures. *Langmuir* **2014**, *30*, 1778–1787, doi:10.1021/la404718e.

2. Guzmán, E.; Ritacco, H.; Ortega, F.; Svitova, T.; Radke, C.J.; Rubio, R.G. Adsorption Kinetics and Mechanical Properties of Ultrathin Polyelectrolyte Multilayers: Liquid-Supported versus Solid-Supported Films. *J. Phys. Chem. B* **2009**, *113*, 7128–7137, doi:10.1021/jp811178a.
